# Future biomaterials for enhanced cell–substrate communication in spinal cord injury intervention

**DOI:** 10.4155/fsoa-2017-0130

**Published:** 2017-11-15

**Authors:** Pradeep Kumar

**Affiliations:** 1 Wits Advanced Drug Delivery Platform (WADDP) Research Unit, Department of Pharmacy & Pharmacology, School of Therapeutic Sciences, Faculty of Health Sciences, University of the Witwatersrand, Johannesburg, South Africa

**Keywords:** biomaterials, extracellular matrix, regenerative medicine, spinal cord injury, stem cells

Neuro(bio)platforms, the extracellular matrix mimicking support structures formed and fabricated from biomaterials, constitute an inherent component of the regenerative strategies employed in neuro-trauma and -degeneration interventions. The choice of these bioplatforms is dependent on several physico-chemical and -mechanical characteristics of the constituent biomaterial(s) which in turn defines the intent (biocompatibility) and extent (biodegradability) of their stay in the neuroenvironment. These factors and features are further essential to realize the complex neuromimetic network formation capable of inherent axonal regeneration with no external mediation. This paradigm develops into an even more complex setting when such archetypes are loaded and seeded with biofactors and stem cells, respectively, and are commonly referred to as ‘combinatorial therapies’ [1]. Within these combinatorial platforms, there is an essential need of effective communication between the host substrate and the resident cells, with the latter building their own home at the expense of the former over an extended neuroresidence time.

The major cause of communication failure between the implanted stem cells and the biomaterial structure is the absence or loss of cell adherence on the substrate. Caron and coworkers, 2016, reported an ECM-deposited, arginine-glycine-aspartic acid (RGD) functionalized, agarose/carbomer based hydrogel to prevent ‘anoikis’ (cell death because of nonadherence of hMSCs on the hydrogel) and successfully preserved the viability of hMSCs for 9 days *in vivo*. The researchers further reported an innovative soaked up approach for cell loading in lyophilized scaffolds to minimize mechanical damage to the cells [2]. Neuronal regeneration is a slow process and requires long stay and extensive differentiation of the transplanted cell within the bioplatforms. Even if this is achieved, long-term teratoma formation presents challenges to further glial differentiation. Führmann and coworkers, 2016, incorporated pluripotent stem cell-derived oligodendrocytes into a RGD/platelet-derived growth factor (PDGF-A) modified hyaluronic acid-methylcellulose injectable scaffold (HAMC-RGD/PDGF-A) and reported an approximate 50% decrease in teratoma formation with the transplanted cells showing glial differentiation. Every component of the HAMC-RGD/PDGF-A interacted with the cells with HA interacting with CD44 receptor, RGD providing adhesion, and PDGF-A provided the integration of grafted cells and host astrocytes/axons. However due to the short stay of the biomaterial structure, the full potential of these functionalizations’ were not extracted [3]. In one of the first studies involving synthetic peptides in spinal cord injury (SCI) intervention, Tysseling-Mattiace and coworkers (2008) developed self-assembling peptide amphiphile nanofibers which were inherently bioactive and provided ECM-mimicking (IKVAV) neuroactive epitopes to the cells in the spinal cord. The laminin epitope presented by the cylindrical nanofibers reduced astrogliosis, increased the number of oligodendroglia and improved the behavioral outcome postimplantation, thereby proving the structural and bioactive benefits of peptide amphiphiles [4,5].

The above-mentioned studies presented complex biomaterial structures to achieve cell survival, growth, migration and differentiation. However, modest but bioreactive biomaterials capable of inherent ECM remodeling and repair may provide an alternative to peptide modification of the biomaterials. Chedly and coworkers, 2017, reported implantation of fragmented physical hydrogel suspension of chitosan (Chitosan-FPHS) to a severed spinal cord. The FPHS modulated the inflammatory milieu *via* the recruitment and polarization of ‘alternatively activated’ M2 macrophages and the anti-inflammatory reactants remained detectable for up to 4 weeks as compared with only 1 week in case of the control group. This immunomodulatory activity leads to remodeling and recreation of the neurovasculature preventing axonal retraction and enhancing cell survival [6]. Tissue remodeling was also reported by Slotkin and coworkers, 2017, wherein the researchers implanted poly-lactic-co-glycolic acid (PLGA)-block-poly-L-lysine (PLL)-based bioresorbable scaffolds (complete degradation within 12 weeks) in a nonprimate SCI model. The PLL component of the biomaterial scaffold provided a positively charged matrix capable of attracting and interacting with the native and regenerating neuronal tissue, such as neurofilament positive neural cortical cells, after appositional placement [7]. A similar PLGA–PLL scaffold is now under clinical trial (NCT02138110) and a recently published case study demonstrated safe implantation of the polymer scaffold into an acutely contused spinal cord. The neurological examination confirmed improvement of injury level from T11 AIS grade A to L1 AIS grade C over 3 months; a first such observation postimplantation of a bioresorbable scaffold [8]. Another example of such nonfunctionalized single component or physically blended bioplatform is the NeuroRegen Scaffold™ fabricated as a linearly ordered collagen scaffold developed by Dai and coworkers over a decade ago [9]. The platform has been extensively functionalized over the years with different collagen-binding proteins with the latest been CBD-EphA4LBD and CBDPlexinB1LBD for neutralizing neuroregeneration inhibiting axon guidance molecules ephrinB3 and sema4D, respectively, making the biomaterials inherently bioactive [10,11]. The NeuroRegen Scaffold seeded with human umbilical cord mesenchymal stem cells (hUCB-MSCs) was clinically tested recently in human subjects with chronic complete SCI. The absence of “*infection, perioperative complications, aggravation of neurological status or cancer*” and increased “*finger activity, enhanced trunk stability, defecation sensation and autonomic neural function recovery*” suggested the potential safety and feasibility of cell-laden scaffolds in chronic SCI intervention [12].

Currently there are more than 15 ongoing clinical trials which involve transplantation of stem cells as the primary interventional approach with only few mentioning biomaterial strategies employed in conjugation with the cell therapy [13]. It is evident from the above discussion that bioengineered strategies will dominate the future research and clinical domain in SCI interventions. For the future biomaterials to carry and sustain the stem cells *in vivo*, materials functionalization can be divided into two approaches: adding ECM-mimicking peptide domains to the biomaterials or using ECM-mimicking bioactive peptides as the whole scaffold; and synthesizing inherently bioactive, ECM attracting and tissue remodeling biopolymeric systems. The essential features of a  5-star cell-laden bioplatform of the future should include: proness: immunomodulation of the proinflammatory neuroenvironment to facilitate macrophage recruitment and proregeneration; stiffness: neuromimetic architecture to mechanically fit into the implant site; thickness: a defined porosity for directional diffusion of the factors secreted by the encapsulated cells; stemness: availability of ECM components and peptides to attach and maintain cells for the lifetime of the platform; and timeliness: ability to degrade at a rate perfectly concurrent with regeneration and formation of new tissue. Although there are several biomaterial systems reported in literature partially fulfilling the above requisites, a perfect neuro-bioplatform is yet to be realized. Given the limited options and selectivity of the stem cells, future biomaterials are required to be designed as multifunctional elements with enhanced cell–substrate communication profile.
